# Supplementary material for: DEAD Box Helicase 24 Is Increased in the Brain in Alzheimer’s Disease and AppN-LF Mice and Influences Presymptomatic Pathology
Source: Int J Mol Sci. 2024 Mar 23;25(7):3622. doi: 10.3390/ijms25073622 (PMC11011903; doi:10.3390/ijms25073622)
Supplement: Supplementary file 1 [file ijms-25-03622-s001.zip › Supplemental figure legends.pdf]

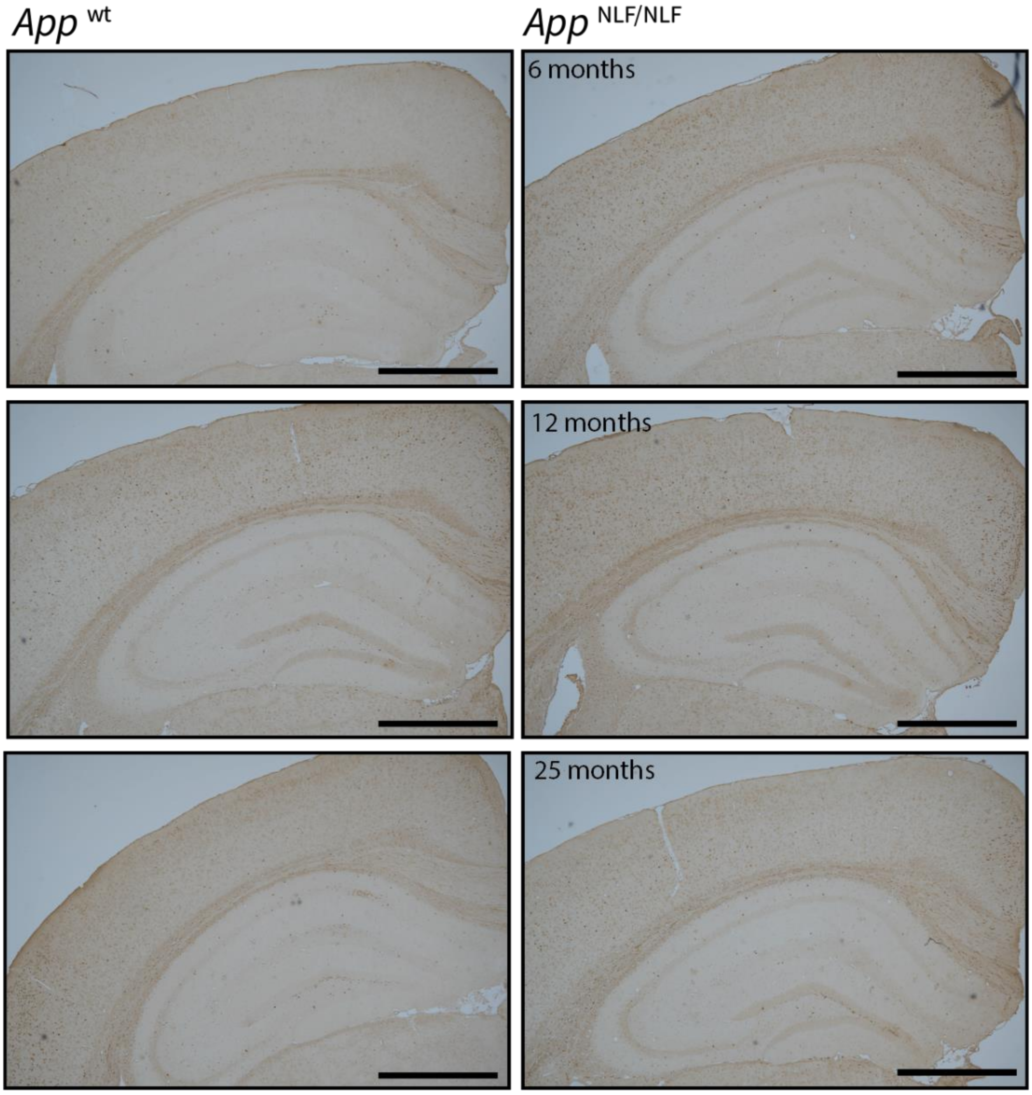

Supplemental figure S1: DDX24 staining in 6 months, 12 months and 25 months old WT and *App* $^{NLF/NLF}$ mice. Scale bar is 150 µm.

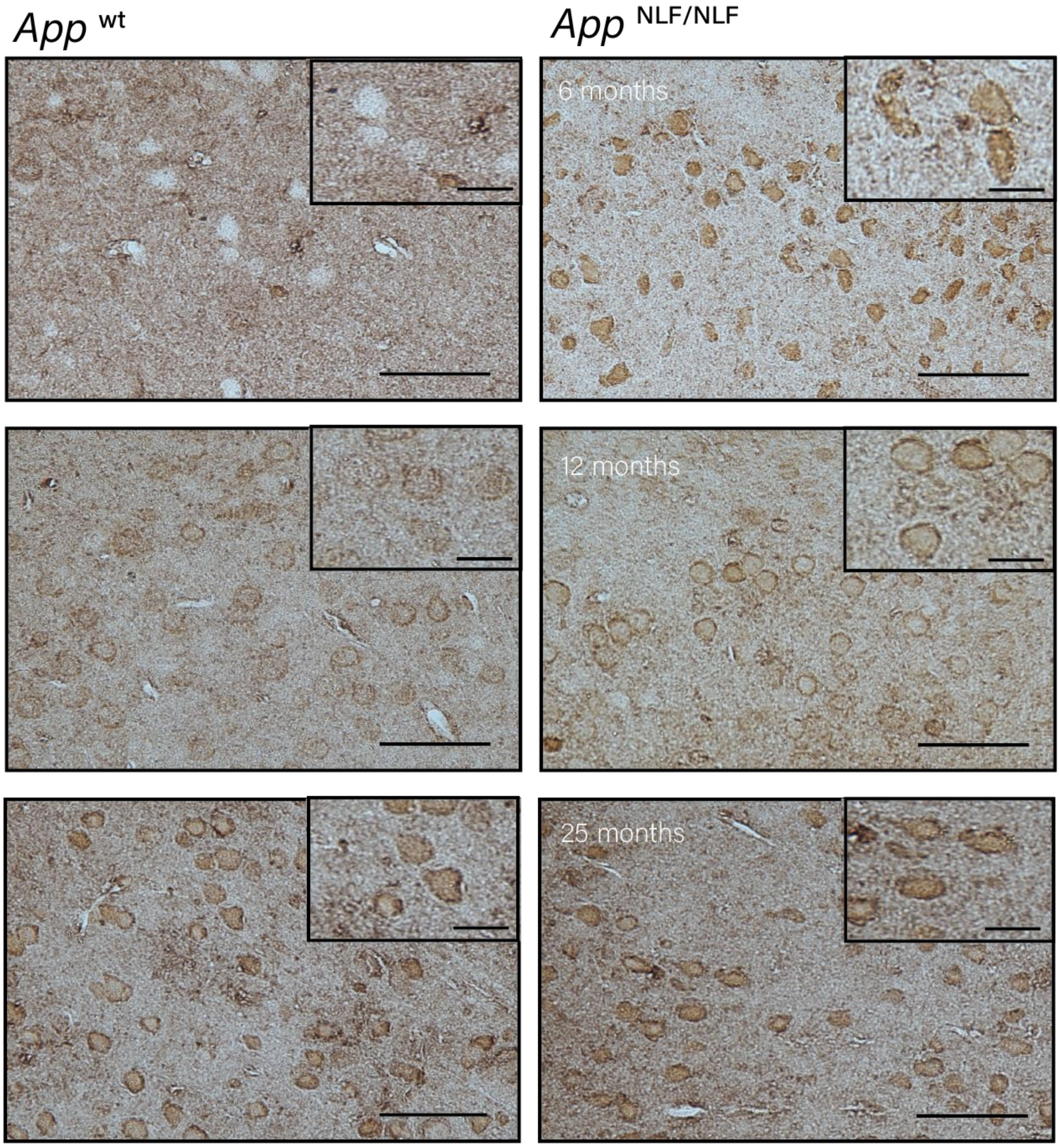

Supplemental figure S2: DDX24 staining in CA3 region of WT and *App* $^{NLF/NLF}$ mice at 6 months, 12 months and 25 months of age. Scale bar is 50 μm in large images and 25 in fold in images.

Supplemental figure S3: Confocal staining of DDX24 in neurites of primary hippocampal neurons derived from WT and *App* $^{NLF\text{-}NLF}$ mice cultured for 7 (A-B), 14 (C-D) and 21 (E-F) DIV. DDX24 is shown in green and phalloidin in white. Scale bars are 5 µm.
