# Supplementary figures and images for: DEAD Box Helicase 24 Is Increased in the Brain in Alzheimer’s Disease and AppN-LF Mice and Influences Presymptomatic Pathology
Source: Int J Mol Sci. 2024 Mar 23;25(7):3622. doi: 10.3390/ijms25073622 (PMC11011903; doi:10.3390/ijms25073622)

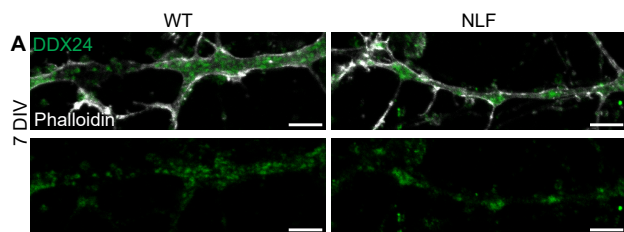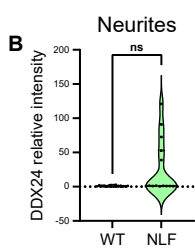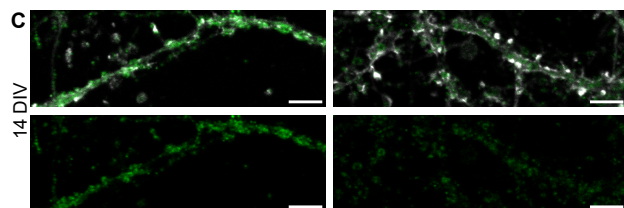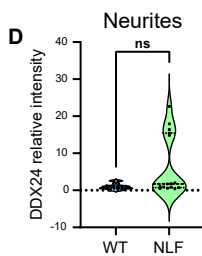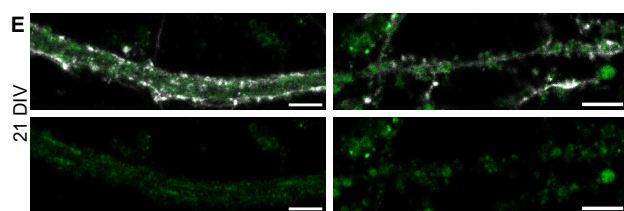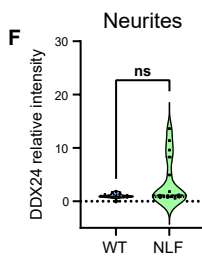

Supplement: Supplementary file 1 [file ijms-25-03622-s001.zip › Supplemental figure S3.pdf]
